# Towards environmental performance through responsible environmental intentions and behavior: Does environmental law cognition really matter among Chinese farmers

**DOI:** 10.1371/journal.pone.0308154

**Published:** 2024-09-06

**Authors:** Ying Wang

**Affiliations:** School of Humanities and Law, Zhengzhou Technology and Business University, Zhengzhou Henan, China; International University - Vietnam National University Ho Chi Minh City, VIET NAM

## Abstract

Rapid agricultural expansion, marked by unsustainable practices, has contributed significantly to environmental degradation globally. In response to China’s escalating environmental concerns, recent environmental legislation has sought to disseminate information and promote awareness of environmental preservation among residents. This study aims to investigate the influential role of citizen environmental intentions and activist environmental intentions on responsible behavior for achieving environmental performance (EP). Moreover, the moderating influence of these environmental laws on the relationship between environment-protecting intentions and behavior, with a focus on legal cognition. Utilizing a population of 3150 farmers we select 603 farmers using simple random sampling and, this study applies the theory of planned behavior within a structural equation model framework. The findings affirm that farmers’ perceived behavior control, personal factors, and attitudes directly impact both citizen and activist intentions, subsequently influencing both types of environmental-oriented behavior. Notably, the study identifies a stronger inward attitude effect compared to the outward attitude in both types of farmers’ intentions. Additionally, legal cognition among farmers emerges as a crucial moderator, influencing the link between environmental intentions and behavior. The results suggest that as farmers become more familiar with environmental laws, the direct effect of their intentions on environmentally oriented behavior intensifies. Hence, the legal obligations play an essential role in shaping the farmers’ pro-environmental behavior. This study aligns the individual level environmental intentions with farmer’s law cognition for offering interesting insights to develop and implement imminent environmental policies. Hence, this study benefits to both practitioners and policy makers’ contextualizing Chinese agricultural sector.

## Introduction

The notion of global warming and emerging changes in the natural environment has gained the attraction of researchers and management of various authorities [1]. These environmental changes exert pressures on societies and business entities to cope with these changes [2]. All kinds of commercial and private entities including agricultural sector facing the regulatory demands for the protection of natural environment [3]. Under these circumstances, societies cannot ignore the strategic importance of environmental performance (EP) for the protection of natural environment [4].

EP is concerned with the practices and operational activities that support the protection and sustainability of natural environment [5]. In the recent decades, EP mechanism become strategic for the researchers and policy makers’ [6]. Protection of natural environment requires responsible ecological supporting behavior and intention [7]. However, the objective of the current study was to explored the individual level factors for the enhancement of EP. In this regard, environmental intention and behavior gained scholarly attention that provide foundational stance for individuals’ to concentrate on pollution reduction and protection of natural environment. The role of individual intention and behavior towards EP is rarely discussed in existing studies on natural environment, however, some empirical findings are available regarding the association among green environmental behavior and EP [8]. Therefore, the current study fill this research gap and aim to explain the role of environmental intention and environmental behavior of farmers’ towards environmental perforance in the context of China.

China is leading grain producer in current polluted world, being the world largest contributor to agriculture, only 10% land area is cultivated [7]. By 2050 the agriculturist will have to feed nine billion people around the world, enhancing the food requirement at least 51%. This shift of rapid enhancement should be foreseen well in advance, focusing on agricultural industry pertinence. Similarly, by 2060, an estimation of 10 billion people in the world’s population will require 25% enhancement in agriculture production. Being the largest agricultural-production, China is feeding 20% population of world and contains only 8% cropland globally [8,9]. The agricultural production capacity belongs the individuals’ intentions and behaviors which degrade sustainability and environmental issues. This study focused on individual intentions and behaviors to shape EP and test the leveraging mechanism of farmer’s law cognition.

The economic strides made by China over the past four decades have, however, been accompanied by significant environmental challenges [10,11]. Despite policymakers and the academic community increasingly prioritizing environmental pollution reduction [6], the nation grapples with severe consequences, including millions of deaths attributed to air pollution [10]. Environmental governance in China relies on planning and law, yet the rapid economic growth has strained the effectiveness of existing regulatory mechanisms [12]. Public awareness of new environmental laws becomes crucial for implementation and ecological accountability. This prompts questions about the potential of law as a tool to govern farmers’ environmental behaviors and whether the rule of law can encourage their active participation in environmental governance [13,14]. Against this backdrop, the goal of this research is to examine the complex interactions that exist between farmers’ environmental behavior, intentions, and overall influence on EP.

In order to promote sustainable agricultural practices and lessen the negative effects of farming operations on the environment, this study is hopeful that by addressing these important issues, future policymakers and intervention strategies will be informed. Previous studies on environmental law and pollution have predominantly centered around assessing law enforcement challenges [15]. Additionally, existing literature has predominantly focused on jurisprudential, micro- and macroeconomic perspectives, and macro-development, often overlooking the nuanced effects of laws on environmentally oriented behaviors for instance [16,17]. Consequently, the study aims to bridge this gap by investigating the moderating role of environmental laws concerning environment-protecting intentions and behavior, specifically emphasizing farmers’ legal cognition. This research contributes to the existing body of knowledge in several significant ways. Firstly, it pioneers an examination of the impact of new Chinese environmental protection regulations on farmers’ environmentally oriented behavior, a fact that has remained largely unexplored in prior studies. Secondly, the study adds practical meaning and social value by evaluating the realistic impact of environmental laws in rural areas, considering resident participation and multi-governance in public administration. To enhance the theoretical model based on the theory of planned behavior and norm activation model (NAM), the research introduces a novel distinction in farmers’ environmental attitudes, separating them into inward and outward attitudes based on daily activities, including farming. Inward environmental attitude reflects individual consumers’ perspectives on environmental issues, while outward environmental attitude pertains to the collective perception that diverse societal aspects necessitate collective action for environmental protection. Furthermore, this study extends its implications globally, particularly for emerging countries grappling with similar environmental challenges and nations under authoritarian regimes. The delicate balance between economic growth and environmental conservation is a critical concern for rapidly growing emerging economies.

The current study provides valuable insights and considerations for encouraging public involvement in environmental conservation, especially among rural residents. Notably, the findings highlight the significant influence of legal cognition on environmental behaviors, urging policymakers to prioritize legal considerations. In sum, this research not only addresses a research gap by examining the impact of new environmental regulations on farmers’ behavior but also contributes practical insights that extend to global environmental challenges and considerations for nations with authoritarian governance structures. By integrating these findings into the theoretical model, this study aims to enrich the understanding of the complex relationships between environmental intentions, behavior, and the moderating role of laws within the agricultural sector.

## Material and methods

### Theory of planned behavior

The theory of planned behavior (TPB) contends that planning of an individual determines the behavior [18]. This theory has been extensively used in various fields, such as public affairs, marketing, sustainable development, sports and health on the association among views, attitudes, intentions, and behaviors [19]. TPB is a psychological model that is used to predict and understand human behavior [20]. In the theory Ajzen proposed that an individual’s intentions are the primary predictors of their behavior, which is based on three main factors i.e. attitude towards the behavior, subjective norms and perceived behavior control [20]. Furthermore, these three factors play foundational role for the intention of an individual to perform a specific behavior. Attitude towards behavior is concerned with the positive or negative evaluation regarding the performance of a particular behavior [18]. According Guerin and Toland [21] academic database contained more than 1200 research references on TPB that supports the stance of researchers for explaining the respondent’s behavior in different context. Moreover, researchers also utilized the concept of TPB to understand human environmental behavior [19,22].

The second elements of TPB i.e. subjective norms are concerned with the perceived social pressures from the members of society, regarding whether an individual should engage in a particular behavior [19]. Subjective norms encompass beliefs about whether important others approve or disapprove of the behavior and the individual’s motivation to comply with these perceived social expectations [20]. The third element perceived behavioral control reflects an individual’s belief in their ability to successfully perform the behavior [20]. It includes perceptions of the ease or difficulty of performing the behavior. TPB suggested that stronger intentions of an individual align with more responsible behavior [22].

Rollon [23] classified behavior in to three categories such as environmental activist behavior, green buying behavior and good civic conduct, which includes diverse types of activities that are essential and beneficial to the environment. The current study is based on the assumptions of TPB that elucidates how farmers’ activist environmental intentions shape their behavior. It emphasizes that strong intentions to engage in environmental activism, influenced by attitudes, social norms, and perceived control, drive farmers to actively participate in advocacy, campaigns, and policy initiatives for environmental causes [21]. TPB posits that robust intentions correlate with increased engagement in activist environmental behavior among farmers [20].

### Norm activation model

Norm activation model (NAM) was developed by Schwartz [1977] to explain altruistic, citizenship and pro-environmental intention and behavior [24,25]. According to the assumptions of NAM, citizenship intentions and behaviors are a function of personal norms set by two factors such as individual ascription of responsibility and awareness of consequences [24,26]. Ascription of responsibility factor of NAM indicates responsibility feelings of an individual for the consequences of pro-social acts [27]. On the other hand, awareness of consequences is concerned with the positive impacts on others as a result of pro-social action [27]. Applying the NAM for the responsible behavior of farmers towards the protection of natural environment is a positive consequence of responsible citizen environmental intentions and activist environmental intentions [26,28]. The current study explained the outcomes of human behavior such as individual intention and behavior. This relationship is best explained in the light of TPB and NAM.

### Relationship between responsible citizen environmental intentions and responsible citizen behavior

Individual awareness regarding natural environment and requirement necessary for the green environment play foundational role for the responsible environmental intention [28–30]. When effectively communicated and embraced by farmers, these intentions can significantly influence and enhance their responsible citizen behavior [31]. On the other hand, individual citizen behavior is based on a myriad of actions, from reducing personal carbon footprints to actively supporting environmental initiatives and activism [32]. These behaviors include energy conservation, waste reduction, pollution reduction and participation in ecological missions [33]. While individual actions may seem modest in isolation, collectively, they exert a significant influence on environmental outcomes [30]. The responsible citizen behavior largely based on the significant knowledge and awareness of green environment as well as the collaboration with various stakeholders, where interaction with stakeholders for accessing the potential needs of their environmental stance [34].

Individual with higher responsible environmental intention contribute for the protection of natural environment, when they think strategically about the green environment requirements [33]. Responsible citizen environmental intention efforts of the individual are necessary for achievement of green environmental behavior [22,26]. Responsible citizen environmental intention directs and facilitates the individual to carry out the environmental activities to fulfil the stakeholder’s demands regarding natural environment. The green environmental stance of individual has significant effects on the responsible citizen behavior regarding the protection of natural environment [29].

One of the major rewards of green environmental intention is it provides awareness and direction regarding the natural environment particularly about existing and potential demands of green environment [22]. Awareness and citizen environmental intention set long term environmental behavior for the protection of natural environment and pollution reduction. The interconnected relationship between conscientious citizen environmental intentions and the conduct of farmers is a pivotal aspect of fostering EP. Primarily, awareness and education regarding the protection of natural environment play significant role in aligning farmers’ behaviors with responsible citizen environmental intentions [28]. Workshops and training sessions relating to environment provide farmers with insights into the ecological consequences of their actions. In line with these arguments, this study formulated that responsible citizen environmental intentions exert powerful influence on farmer’s behaviors. By promoting awareness, offering incentives and accelerating the convergence of these intentions with agricultural practices can lead to a paradigm shift towards green farming [29,30]. Encouraging farmers to embody responsible citizen behaviors not only ensures the viability of agricultural livelihoods but also safeguards the environment for generations to come.

*H1 Environmental intentions predicts citizen behavior of farmers*

### Activist environmental intentions and behavior

Activist environmental intention is concerned with the proactive and passionate drive of an individual towards advocating for environmental causes [35]. Simply activist environmental intention is the willingness of an individual to engage in green operational activities that support the protection of natural resources [36]. Activist intentions regarding the protection of natural environment can be considered as an integral part of individual behavior to enhance the EP [37]. When these intentions are effectively embraced by farmers, they can significantly influence and enhance their activist environmental behavior, leading to a profound impact on sustainable agriculture. Farmers with green environmental intention are more inclined to reduce the pollution and waste material that is necessary for the improvement of environmental behavior [31,38]. Activist environmental intentions play foundational role for increased awareness among farmers regarding the environmental issues and protection of natural environment [39]. Exposure to information regarding broader environmental challenges, such as climate change, biodiversity loss, or soil degradation, inclines farmers towards adopting activist roles. Farmers with higher level of activist environmental intentions are more likely to engage in green environmental efforts, participate in policy discussions and contribute for green farming practices.

Activist intentions towards green environment and community engagement improve the farmer’s activist behavior necessary to minimize the harmful material and emission that is the outcomes of farming process [31]. Recognizing that their collective efforts contribute to a larger movement towards environmental sustainability strengthens their resolve to actively participate and advocate for change. In conclusion, the embrace of activist environmental intentions among farmers has the potential to significantly impact their behavior towards more environmentally conscious practices [35].

Existing studies documented that individual with green environmental intention about the stakeholder’s environmental demands increases the movement and action for environmental conservation and farming practices [22,25,26]. Activist environmental behavior mechanism of an individual is successfully promoted with higher level of green environmental intention and desire regarding the protection of natural environment. In line with TPB this study suggested that farmer’s environmental intention play important and foundational role for demonstrating the green environmental behavior.

Hypothesis 2:

H2 Activist environmental intentions predict environmental behavior.

### Environmental citizen behavior and EP

Responsible citizen behavior consists of individual actions, from reducing pollution to actively supporting and encouraging green environmental activities [36]. These actions play important role for the improvement of EP which is necessary for the protection of natural environment [40]. Individual actions and decisions have major influence for his or her participation in green environmental practices [22]. One dynamic impact of environmental citizen behavior is its role in driving demand for eco-friendly products and services [41]. As more individuals prioritize sustainability in their consumption patterns, businesses respond by offering environmentally friendly alternatives [42]. This shift in consumer preferences often leads to improvements in product design, production processes, and supply chain management, fostering a more sustainable marketplace. Moreover, citizen behavior influences policy agendas and societal norms [36].

Existing studies empirically found that citizens, societies and multiple institutional pressures stimulate regulatory authorities to formulate green environmental practices that ensure the green environment through the reduction of pollution [31,40]. The societal participation contribute to shaped regulations and regulatory frameworks, contributing to advancements in EP at both national and global levels [41]. EP refers to measurable outcomes related to resource conservation, pollution reduction, and overall environmental health [43–45]. The influence of citizen behavior on EP can be observed through indicators such as carbon emissions, waste generation, biodiversity preservation, and energy consumption [36,46]. According to [41,47] the impact of environmental citizen behavior of individual is strongly related with EP. Awareness and economic sustainability encourage the environmental behavior which is necessary for the protection of natural environment [26,48].

Zmihorski et al. [49] suggested that individual with higher levels of environmental behavior significantly contribute for better EP. Reduced carbon footprints, increased recycling rates, preservation of green spaces, and the adoption of sustainable energy sources are among the outcomes associated with proactive citizen engagement in environmental sustainability efforts [17]. In line with these arguments, this study formulated the following hypothesis:

H3 Environmental Citizen Behavior positively predicts EP

### Activist environmental behavior and EP

Activist environmental ways of behaving cover a range of actions, from coordinated fights and missions to campaigning for strategy variations and participating in common noncompliance [31]. Such a way of behaving is in many cases propelled by a need to get a move on to address environmental problems like environmental change, biodiversity misfortune, and contamination [34]. Activists endeavor to carry problems to light, incite exchange, and push for unmistakable answers for environmental problems. One of the most well-known effects of activists’ environmental ways of behaving is its capacity to influence strategy-making and institutional variation [36]. Through promotion and public pressure, activists force states and establishments to take on and uphold harmless ecosystem arrangements [32]. Models remember guidelines for discharges, security of regular environmental surroundings, and interests in sustainable power, all of which straightforwardly influence EP poles [33].

Besides, activist ways of behaving habitually serve as an incentive for cultural variation. By stirring things up and featuring environmental treacheries, activists invigorate public talk and change cultural standards [35]. These prompts expanded mindfulness, moving social mentalities toward manageability, and advancing capable utilization and protection endeavors. Furthermore, activist environmental way of behaving rouses advancement and cultivates the development of feasible performs [31]. By bringing problems to light of natural issues, activists animate exploration and mechanical progressions in sustainable power; squander the executives, and asset productivity. This development adds to worked on EP across businesses [36]. Nonetheless, the effect of activist environmental conduct on EP faces problems and reactions. Some contend that limits or fierce strategies utilized by activists might estrange partners or the overall population, possibly thwarting more extensive help for environmental causes [39]. Also, completing agreement and making an interpretation of activism into long-term strategy variations can be complicated and tedious.

EP refers to the quantifiable results and moves made to relieve natural effects. This incorporates poles like fossil fuel by-products, energy utilization, squander age, and preservation endeavors [6]. The influence of activist environmental behavior on EP can be understood through variations in these measurements. Areas or networks that are key areas of strength with commitment habitually display better EP poles [15]. Diminished carbon impressions, expanded reception of environmentally friendly power, higher reusing rates, and the protection of biological systems are results related with proactive activist way of behaving focused on environmental supportability [19]. All in all, an activist environmental way of behaving fundamentally influences EP by creating strategy plans, cultural standards, cultivating advancement, and motivating aggregate activity [26]. While confronting problems, the constant endeavors of activists keep on driving positive variation, adding to a more possible future for the planet.

*H4 Activist Environmental Behavior positively predicts the EP*

### Moderation of environmental laws between citizen environmental intentions and behavior Hypothesis 7a

The current study considered the moderation of environmental laws on the connection between responsible citizen environmental intentions and responsible citizen behavior. Study hypothesis 1 shows that responsible citizen environmental intentions have positive role for the responsible citizen behavior. The interplay between responsible citizen environmental intentions and the behavior of farmers definitely holds significant sway in shaping sustainable agricultural practices [13]. Environmental laws, functioning as regulatory frameworks and guidelines, play a crucial role as positive moderators, reinforcing this relationship by establishing a structured foundation and fostering conducive environment for responsible behavior among farmers [50]. Responsible citizen environmental intentions embody the proactive mindset and conscious efforts individuals undertake for the protection of environmental [20]. When these intentions are synergized and complemented by robust environmental laws, they become instrumental in steering farmers towards responsible behavior [51]. Environmental laws induce farmers for the adoption of green practices that ensure the protection of natural environment [43]. These laws serve as a cornerstone in influencing farmers’ behavior by establishing clear standards and expectations for environmental stewardship within the agricultural sector [17,21]. Moreover, environmental laws offer a legal and ethical directions and foundation that exert pressures on farmers to involve in responsible environmental behavior [43,44]. Compliance with environmental laws encourages the farmers towards environmental preservation [17]. The awareness that their actions are governed by legal mandates encourages farmers to internalize responsible citizen environmental intentions and integrate them into their farming practices [14]. Environmental laws also play significant role for raising awareness and knowledge about the importance of environmental issues [16]. This educational aspect reinforces responsible citizen environmental intentions by providing farmers with the necessary knowledge and tools to make informed decisions aligned with environmental conservation [12]. Ultimately, the synergy between responsible citizen environmental intentions and the supportive framework of environmental laws paves the way for a more conscientious and environmentally responsible farming community.

*H5 Environmental laws positively moderate on the connection between responsible citizen environmental intentions and responsible citizen behavior*.

### Moderation of environmental laws between activist environmental intentions and behavior

Connection between activist environmental intentions and responsible activist behavior of farmers plays important role in steering agriculture sector for green environmental practices [22]. This connection is positively moderated by environmental laws that are functioning as regulatory mechanisms and induce the farmers to convert their activist environmental intentions in to environmental proactive behavior [24]. Farmer’s activist environmental intentions plays important role for proactive behavior regarding the protection of natural environment [26]. When farmers’ activist intentions are reinforced and complemented by robust environmental laws, it creates a conducive environment for them to engage in activist environmental behavior within their farming practices [23,52].

Environmental laws are the guidelines and regulations for the execution of farming activities that support the green environment [43]. These laws stipulate regulations regarding land use, conservation practices, emission standards, waste management, and other critical facets of farming that directly impact the environment [17]. Environmental laws provide a framework and parameters within which farming activities are performed that ensure the green environment [52]. Environmental laws increase the farmer’s environmental intentions and behavior towards the protection of natural environment [23]. Farmer’s having environmental intentions along with directions and guidelines of environmental laws shows higher proactive environmental behavior [22]. Environmental laws also play a crucial role in fostering awareness and education among farmers about the significance of activist environmental behavior [53–55]. In line with these arguments this study suggested that activist environmental intentions in the presence of environmental laws strongly support the activist environmental behavior.

H6 Environmental laws positively moderate on the connection between activist environmental intentions and responsible activist behavior.

### Theoretical framework

**Fig 1:**
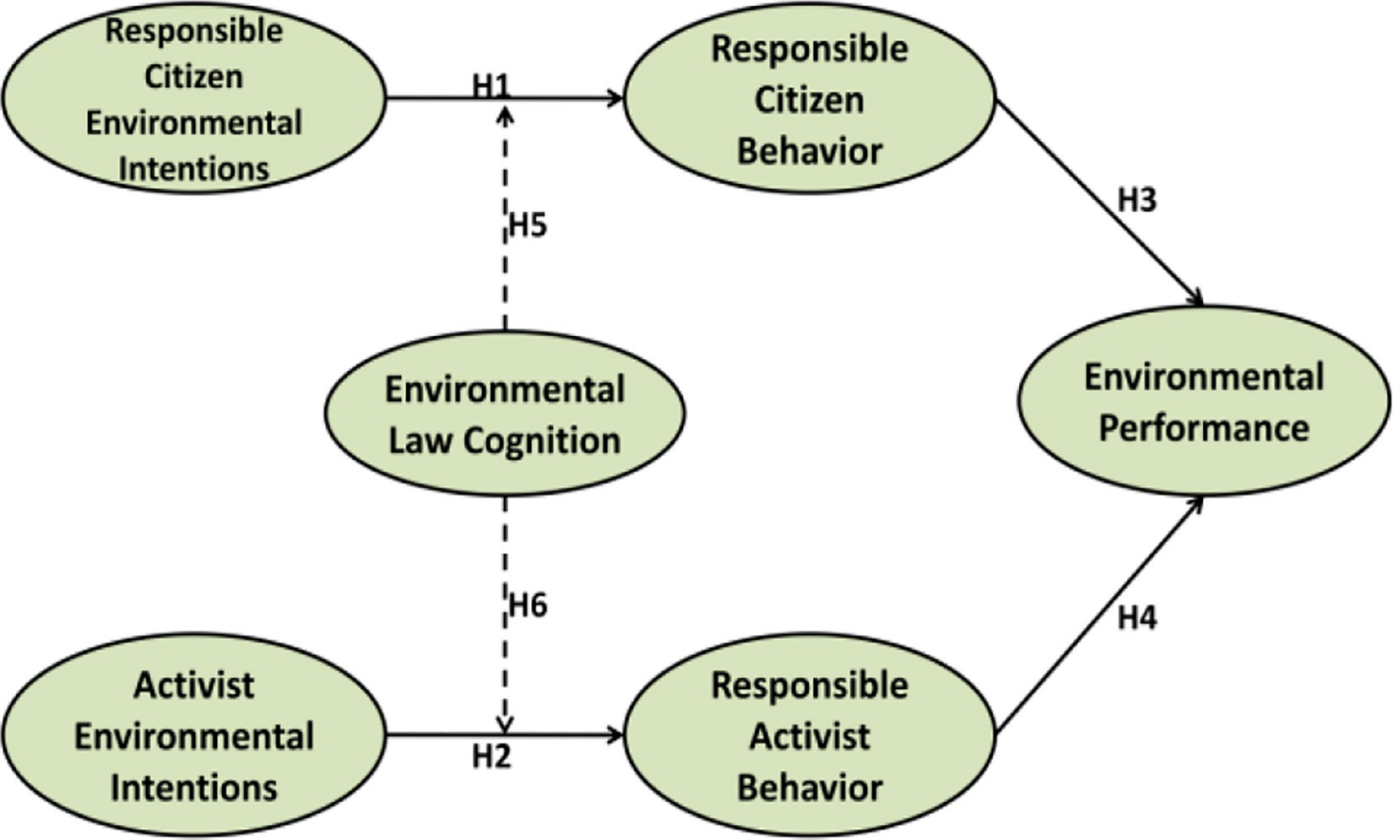
Theoretical framework.

Fig 1 presents the association among study constructs. This study used six variables i.e. responsible citizen environmental intention, activist environmental intention, responsible citizen behavior, responsible activist behavior, environmental law cognition and EP. Fig 1 is used to show the direct path from responsible citizen environmental intention and responsible citizen behavior. Fig 1 also shows the direct path from activist environmental intention and responsible activist behavior. Furthermore, moderating role of environmental law cognition also depicted in (Fig 1).

## Methodology

### Study area

Jiangxi Province, situated in the southern part of China along the south bank of the Yangtze River, encompasses a land area of 166,900 km2 and is home to over 46 million residents. Positioned at 24°29’–40°40’ N and 113°34’–118°28’ E, the province boasts a subtropical monsoon climate characterized by an average annual rainfall of 1700–1943 mm and an average annual temperature ranging from 16.3–19.5°C. Renowned as a major grain-producing region in southern China, Jiangxi has a total grain cultivation area of 36,651 hectares, yielding an annual grain output of 21.574 million tons. Notably, grain crops constitute 60 to 70 percent of the total crop area, with rice occupying over 85 percent of the acreage allocated for grain cultivation. The province is an agricultural stronghold, primarily engaged in the production of essential items such as grain, oilseeds, vegetables, and aquatic products, positioning it as a prototypical agricultural province and one of China’s 13 key grain-producing provinces [56].

### Survey design, sampling and data collection

It is a cross-sectional study and questionnaire is the main tool used for data collection, which is kept as concise as possible with simple/easy to understand wording [57]. The major strength of cross-sectional studies is that their results can be generalized to the whole population from the base of some small representative sample. It is reliable and valid to make conclusions regarding the issue being addressed in the study paving way for others to use it as reference for future studies [58].

To attain the objectives, the current study used cross-sectional research design which entailed collecting quantitative data from respondent at a single point in time. The current study is quantitative based which anchored on quantitative observable reflections. Through quantitative research this study measures the phenomenon by answering the questions related to how much, how many, how often, when and who in numeric terms. The survey method is adopted for data collection from the respondents. The target population consists of farmers of China. A list of 3150 farmers was finalized as a target population. Through simple random sampling the study selects 603 farmers for the purpose of data collection. For data collection the services of 4 research assistants were hired. During data collection process only 580 questionnaires were received back from farmers.

To ensure a comprehensive representation of China’s agricultural landscape, a multistage purposive and random sampling technique was employed to gather data from farmers in Jiangxi Province. Initially, Jiangxi was purposively selected due to its pivotal role in Chinese agriculture. Subsequently, three strategically chosen cities namely, Jiujiang, Nanchang, and Ganzhou were selected to enhance the statistical representativeness of the sample across the northern, middle, and southern regions of the province. Further sampling involved the random selection of five counties from each city and, subsequently, the random selection of five villages from each county. Random selection of sampling reduces the chances of sampling bias, because this method ensures the selection of sample strictly by chance [24,59]. The final step encompassed the random selection of twenty-five farmers from each village, resulting in a total of 580 valid responses. The survey, executed by a team of ten proficient researchers, both male and female, ensures the reliability and relevance of the collected data within the context of our theoretical framework. Conducted through cross-sectional data analysis, the current research utilized a meticulously crafted questionnaire with written informed consent to investigate the myriad factors influencing the environmental behavior of farmers or rural residents. Notably, the questionnaire underwent a thorough refinement process, wherein the study specifically identified potential factors impacting farmers’ behavior. Before commencing the field survey from 22^nd^ Dec 2022 to 25^th^ July, a two-step process was employed to assess the reliability and validity of the data collection instrument. Initially, four experts specializing in farm-related environmental regulation, farm management and environment studies reviewed the survey instruments, validating their alignment with relevant data and technical terminology [60]. Subsequently, a pilot study involving 40 farmers was conducted to refine the data collection process, ensuring its congruence with the practical realities faced by farmers on the ground [61].

### Measurement

#### Responsible citizens’ environmental intentions

For the measurement of RCEI 6 items scale was used which is adopted from [62]. This construct measures the environmental intentions of responsible citizens by evaluating their allegiance to environmentally conscious behavior and awareness of ecological consequences. The question example is “I feel good to decide generally green tour”.

#### Activist environmental intentions (AEI)

To measure AEI 5 items scale was used which is adapted from [63]. This variable evaluates people’s ardent dedication to promoting environmental causes and their proactive participation in initiatives that advance ecological sustainability. The sample question is “I will not at all permit the any plan or creation that may create the contamination in surroundings”.

#### Responsible citizen behavior (RCB)

The RCB is measured through 7 items scale which is adapted from [64]. This construct assessing an individual’s real participation in sustainable practices and pro-environmental activities which is a necessary part of measuring responsible citizen behavior, or RCP. For example, “I forever utilize the crops extra material at ranch to boost the earth richness”.

#### Activist Environmental behavior (AEB)

The AEB is measured through 4 items scale which is adapted from [64]. The process of quantifying activist environmental behavior, entails determining how much a person has really gone above and beyond in supporting and engaging in environmental causes and sustainable practices. I forever support the agricultural estate at ranch in region is the example item.

#### Environmental law cognition (ELC)

For the measurement of ELC 10 items scale was used which is adapted from the studies of [65]. Evaluating farmer’s knowledge about the restrictions on tailpipe emissions, legal responsibilities, ecological toxic waste etc. and comprehension of environmental laws is known as environmental law cognition (ELC). The sample item is “The rule also describes sound as pollutant”.

#### Environmental Performance (EP)

To measure EP 4 items scale was used which is adapted from [66]. Assessing the observable consequences and outcomes associated with resource preservation, pollution control, and general ecological well-being is part of measuring EP. The sample item is “Environmental activities overcome the lead time”.

## Results

To perform the statistical analysis for collected data, this study used SPSS (statistical package for social sciences) version 23 and SEM. [60,61]. This research applied hierarchal regression for the analysis of moderation.

### Reliability and validity test

Reliability analysis is used for the overall stability and consistency of a measure. Table 1 shows the outcomes of validity test and discriminant and convergent validity results are found to be correct. Reliability and validity of the study constructs and measures were established with the help of Cronbach’s alpha, factor loading and AVE. Table 1 contained the coefficients of Cronbach’s alpha, factor loading and AVE. Reliability of the measures was established as the value of Cronbach’s alpha is above 0.70 that is threshold value for the confirmation of measures reliability. The coefficients of factor loading also confirmed that each factor measured its intended construct. The coefficients of AVE for each constructs indicated good internal consistency of the constructs [61]. Finally, the composite reliability (CR) value which is the outcome of square of factor loadings confirmed the construct reliability as the coefficients are greater or equal to 0.70.

**Table 1:** Results ExamineFac-loading, Alpha CR-Value and AVE-Value.

Construct Details	Items	FL	Value of Alpha	CR	AVE
Responsible Citizen environmental intention	6	0.72–0.84	0.81	0.94	0.72
Activist Environmental intentions	5	0.71–0.86	0.85	0.98	0.76
Environmental law cognition	10	0.74–0.81	0.88	0.96	0.74
Responsible citizen behavior	7	0.71–0.87	0.86	0.92	0.70
Responsible Activist behavior	4	0.76–0.82	0.82	0.90	0.71
Environmental Performance	4	0.73–0.88	0.84	0.91	0.78

Table 2 specifies the results of correlation matrix, standard deviation and mean value. The VIF scores were less than the cut-off values of 10.0 that verified that there was no matter of mutli-collinearity.

**Table 2:** Correlation matrix.

Sr.No		M.V	S.D	1	2	3	4	5	6	7	8
1	Farmer Age	1.24	0.46	1.00							
2	Farmer Education	1.47	0.47	0.147**	1.00						
3	Responsible citizen environmental intention	3.78	0.52	-0.04	0.05	1.00					
4	Activist Environmental intentions	1.48	0.58	0.547	0.07	-0.16	1.00				
5	Environmental law cognition	1.78	0.67	0.587	-0.04	-0.02	-0.03	1.00			
6	Responsible citizen behavior	1.43	0.63	0.578	0.02	-0.078	-0.03	0.32*	1.00		
7	Responsible Activist behavior	1.51	0.61	-0.06	0.06	0.035	0.26*	0.34*	0.18*	1.00	
8	Environmental performance	3.47	0.68	-0.06	-0.01	0.02	0.28*	0.20*	0.24*	0.16*	1.00

Note

* = <0.005

** = <0001.

### Structural equation modelling

Structural equation modelling (SEM) is one of the emerging techniques for assessing the hypothesized model [43]. With the help of SEM researcher can analyzed data with variance based approach (PLS-SEM) and covariance base analysis (CB-SEM), the current study applied PLS-SEM approach in order to explained variance of dependent constructs. it consists of path analysis having latent constructs for the measurement of causal relationships. SEM is based on two-step approach i.e. structural and measurement model.

### Measurement model

Measurement model which is consist of six factors including responsible citizens environmental intentions, activist environmental intentions, responsible citizen behavior, activist environmental behavior, environmental law cognition and environmental performance. CFA is used to analyzed the measurement model of the current study.

### Confirmatory factor analysis

Confirmatory factor analysis-CFA-model presents the model fitness see Table 3 and results found to be correct. Factor 6^th^ factor model shows the model fitness χ2 = 918.57, Df = 375, χ2/Df = 2.450, RMESHA = 0.048, GFI = 0.97, CFI = 0.96.

**Table 3:** Results of CFA.

Model Description	χ2	Df	χ2/Df	Rmesha	GFI	CFI
6-FM	918.57	375	2.450	0.048	0.97	0.96
5-FM	987.25	370	2.668	0.046	0.87	0.86
4-FM	1025.69	315	3.256	0.042	0.77	0.76
3-FM	1134.42	355	3.196	0.038	0.67	0.66
2-FM	1245.81	350	3.559	0.036	0.57	0.56
1-FM	1287.25	345	3.731	0.028	0.47	0.46

FM = factor model.

### Structural model

The structural model as a part of SEM represents the connection among the constructs of hypothesized model. In the structural model various paths shown association between exogenous (independent) and endogenous (dependent) constructs used the theoretical framework developed on the basis of literature review. Table 4 shows the results of various paths of structural model.

**Table 4:** Results of structural model regarding research hypothesis.

Hypothesis	Relationship	Beta-V	Std. Dev	Decision
H1	RCEI→RCB	0.46	0.044	Proved
H2	AEI→RAB	0.41	0.052	Proved
H3	RCB→EP	0.44	0.048	Proved
H4	RAB→EP	0.48	0.082	Proved

Source: Results Analysis.

Table 4 presents the results of hypothesis using structural equation modelling (SEM). H1 propose that RCEI predicts RCB (B = 0.46, p = sign), H1 is accepted. H2 directed AEI positively associated with RAB (B = 0.41, p = sign), hence H2 is accepted. Similarly, RCB positively predicts EP (B = 0.44, p = sign), hence H3 is accepted. Hypothesis H4 proposes that RAB positively associated with EP (B = 0.48, p = sign), hence H4 is accepted.

### Analysis of moderation

**Fig 2:**
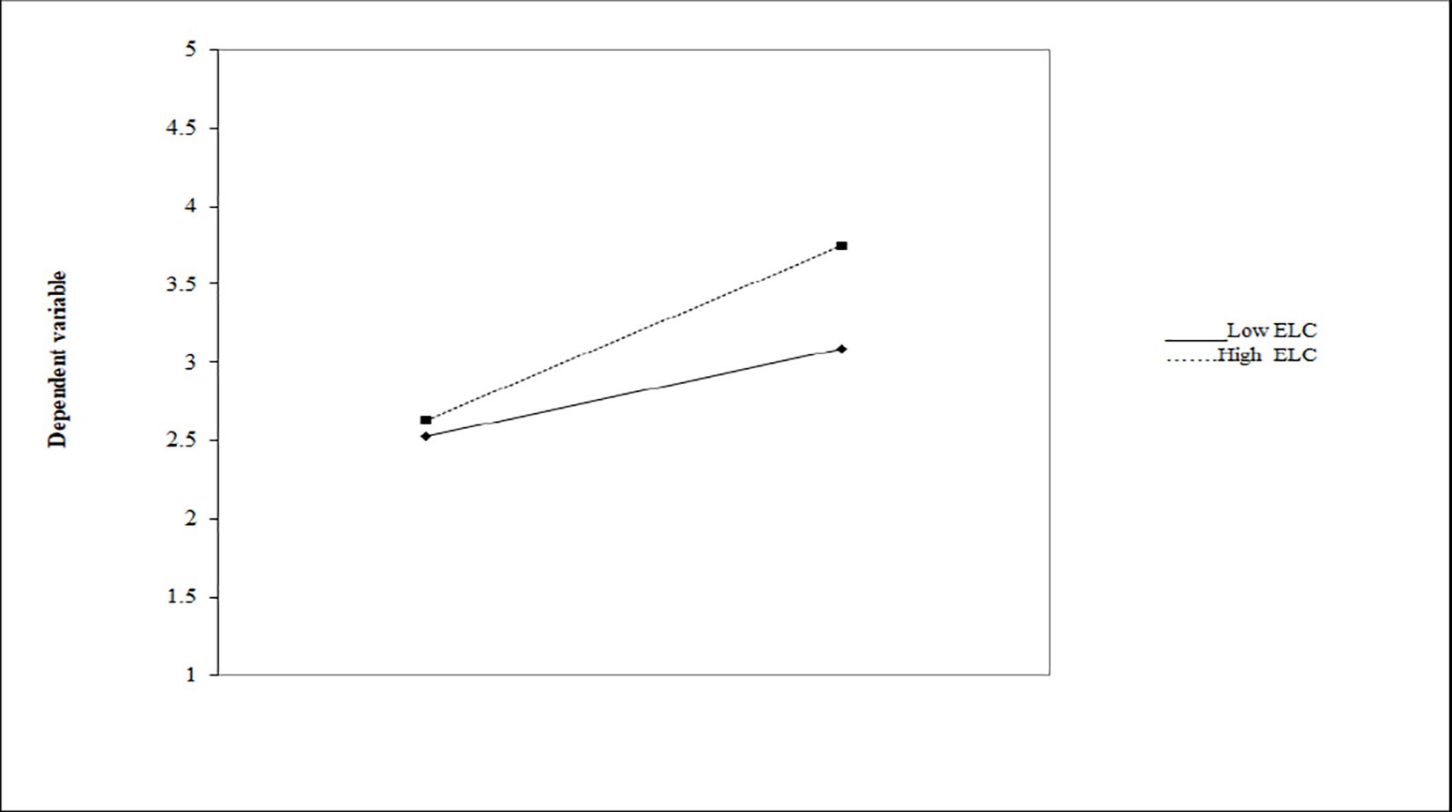
Moderation effect of environmental law cognition on responsible citizen environmental intention and responsible citizen behavior.

Hierarchal regression analysis was used to test the moderating effect of environmental law cognition (ELC) between responsible citizen environmental intention (RCEI) and responsible citizen behavior (RCB). Results show the direct effect and significance influence see (Table 5) on environmental law cognition (β = 0.11, p < 0.01) and responsible citizen environmental intention (RCEI), (β = 0.36, p < 0.01) on dependent variable i.e, farmer’s responsible citizen behavior (RCB). Similarly, the interaction influence of ELC and RCEI had positive and significance predict role on responsible citizen behavior i.e, (β = 0.41, p < 0.01). Therefore, this research delivers practical evidence that ELC moderates the impact of responsible citizen environmental intention on responsible citizen behavior. Hence H5 is accepted. Slop analysis show the moderating effect of environmental law cognition between RCEI and responsible citizen behavior in (Fig 2).

**Table 5:** Moderating role of environmental law cognition between RCEI and RCB.

Research Variables	Co-efficient	SE	R	R2	Value of F
Environmental law Cognition	0.11	0.030*			
RCEI	0.36	0.046*	0.48	0.23	42.84*
ELC x RCEI	0.41	0.038*			

Note: RCB is dependent variable

*depicts significance1%

**Fig 3:**
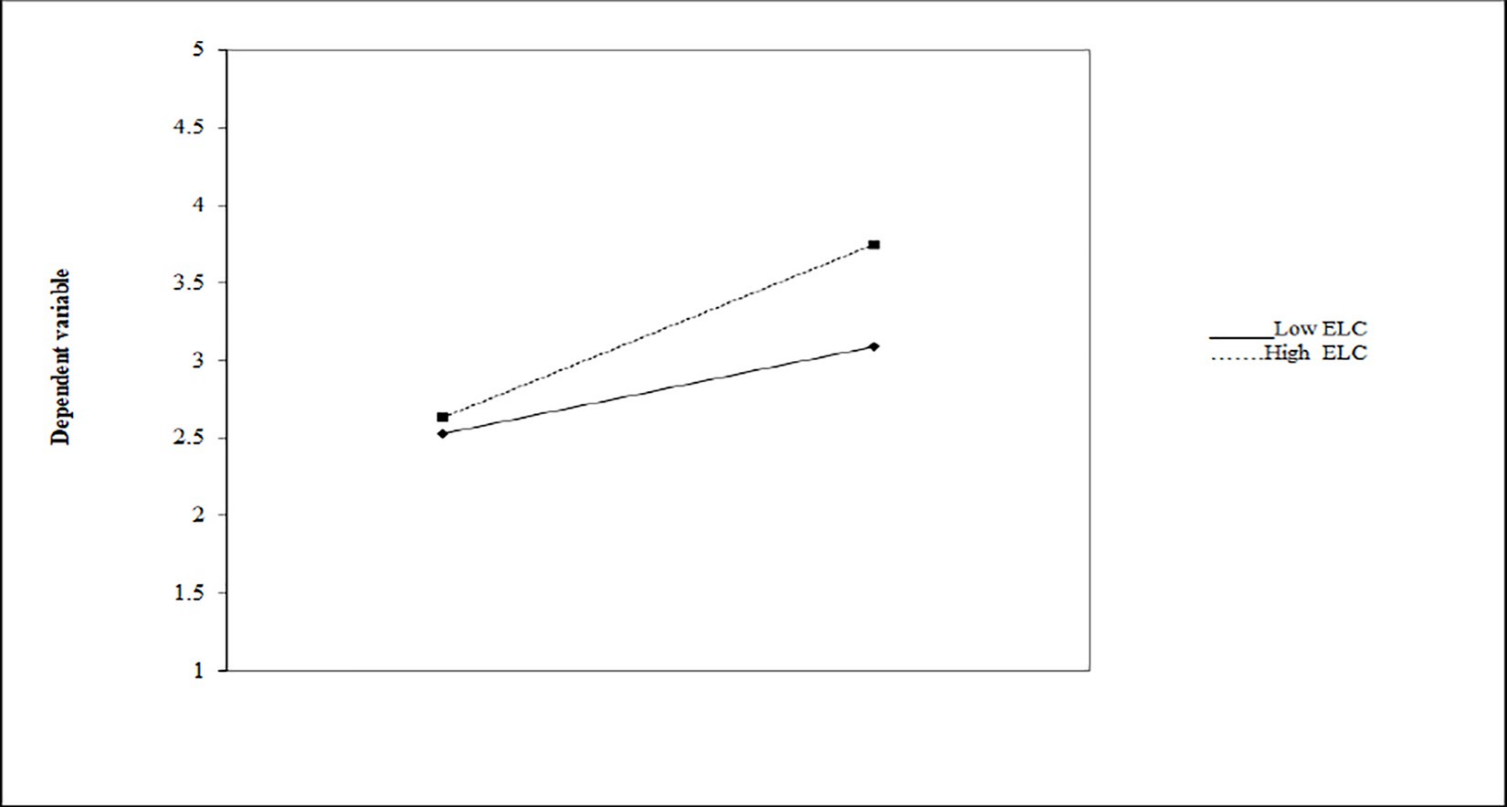
Moderation effect of environmental law cognition on activist environmental intention and responsible activist behavior.

Hierarchal regression analysis was used to test the moderating effect of environmental law cognition (ELC) between activist environmental intention (AEI) and responsible activist behavior (RAB). Results show the direct effect and significance influence see (Table 6) on environmental law cognition (β = 0.10, p < 0.01) and activist environmental intention (AEI), (β = 0.34, p < 0.01) on dependent variable i.e., farmer’s responsible activist behavior (RAB). Similarly, the interaction influence of ELC and AEI had positive and significance predict role on RAB i.e, (β = 0.40, p < 0.01). Therefore, this research delivers practical evidence that ELC moderates the impact of activist environmental intention on responsible activist behavior. Hence H6 is accepted. Fig 3 also explains and presents the moderating effect environmental law cognition between activist environmental intention and responsible activist behavior.

**Table 6:** Moderating role of environmental law cognition between AEI and RAB.

Research Variables	Co-efficient	SE	R	R2	Value of F
Environmental law Cognition	0.10	0.029*			
AEI	0.34	0.044*	0.47	0.22	41.83*
ELC x AEI	0.40	0.037*			

Note: RAB is dependent variable

*depicts significance 1%.

## Discussion

The dynamic environment of agriculture sector of China has a high preference for the achievement of EP. The current study proposed the connection among responsible citizen environmental intention, activist environmental intention, responsible citizen behavior, responsible activist behavior, environmental law cognition and EP. The findings suggested that responsible citizen environmental intention have positive connection with the responsible citizen behavior of farmers. The findings also confirmed the positive effect of activist environmental intention towards responsible activist behavior. Furthermore, responsible citizen behavior and responsible activist behavior enhances the EP. Finally, the findings also revealed that environmental law cognition strengthen the connection between responsible environmental intentions and behavior for farmers.

This study examined the method that farmers can achieve EP through intentions and behaviors. This research developed an “EP Model for farmers” based on six hypotheses. Results demonstrate H1 on the symbiotic relationship between citizen environmental intentions and the behavior of farmers. The developed assumption concerning H1 was proofed by the knowledge of professionals that farmers can achieve EP by implementing agricultural practices. H1 support the efforts of earlier researchers like [27,28]. Concerning H2, results demonstrated that activist attentions predict the farmer’s behavior. The earlier researchers like [16,33] support the finding and H2 was proved that activist environmental intentions among farmers have the significant impact on their behavior towards more environmentally conscious practices. H3 exposes that citizen’s behavior predicts the EP. The study results proved that citizens behavior play an important role in achieving EP. The study of previous researchers like [39–41] supports the findings that farmers with aim to achieve EP adopt agricultural strategies to save environment. Regarding H4 exposes that activist environmental way of behaving fundamentally influences EP. Achieve EP by creating strategy plans, cultural standards, agricultural advancement, and motivating aggregate activity. The study findings are aligned with prior researchers like [29,42,43,46]. H5 shows that Environmental law moderate citizen environmental intentions and behavior. Citizen environmental intentions and the behavior of farmers play crucial role in achieving EP. Environmental laws provide ways to adopt responsible behavior. The findings are helpful with the earlier researchers like [13,51,53] that environmental laws provide a legal and ethical base that incentivizes farmers to adopt responsible behaviors. Finally, the moderating role of environmental law cognition is tested through H5 i.e. the connection between responsible citizen environmental intentions and responsible citizen behavior. The findings suggested that laws regarding environment positively contributes for raising awareness and knowledge about the importance environmental issues [16]. Consequently, the synergy between responsible citizen environmental intentions and environmental laws paves the way for a more conscientious and environmentally responsible farming community [12,48]. While H6 shows that Environmental laws moderate between Activist environmental intentions and Behavior. Environmental laws, as guiding frameworks, play an important role in making activist environmental intentions into proactive behavior. The findings suggested that farmers with higher environmental intentions towards natural environment along with directions of environmental laws shows greater proactive environmental behavior [22,44]. H6 support the efforts of earlier researchers like [39,47,49]. The findings show an addition in the earlier researcher’s research that intentions and behavior has a direct link with EP.

### Theoretical implications

This study has made a greater contribution to the existing literature does have several theoretical implications. This research has provided an in-depth understanding of EP of agriculture sector of China by discussing the various perspectives that affects it. At first stance results that are derived from the research, the sample illustrates that if the farmers are environmental oriented, they have a great influence on EP.

As a whole, the proposed hypotheses provide a thorough theoretical framework that helps explain the relationships that exist between individual’s intentions, behaviors, as well as EP in the agriculture industry. Farmers’ intentions toward the environment, especially those of activists, should be encouraged and supported, as the first two set of assumptions (H1 and H2) emphasize. Theoretically, encouraging an enthusiastic and proactive dedication to environmental causes can greatly impact farmers’ daily actions and eventually lead to an additional sustainable farming environment. The concepts (H3 & H4) also highlight the important part that individual actions play in determining the overall performance of the environment. Encouraging ethical and activist environmental attitudes is crucial for improving overall sustainability within agriculture, as the theoretical framework underscores the interdependence between farmer actions and wider environmental outcomes in agriculture industry. Additionally, the modulating factors (H5 as well as H6) pertaining to environmental laws that have been presented significant understanding of the regulatory framework. For the most part, the theoretical framework provides insightful direction for practitioners, researchers, and policymakers who aim to improve environmental stewardship in the agricultural sector.

### Practical implications

The hypotheses being proposed have practical implications that can direct policies and interventions meant to promote environmentally sustainable practices in the agriculture industry. First, H1 indicates that encouraging farmers to have good environmental intentions can be an effective means to affect their daily actions. The necessity of awareness campaigns, educational initiatives, and financial incentives to persuade farmers to embrace environmentally conscious practices is highlighted with the practical implications. Farmers’ behavior may change for the better as a result of initiatives that emphasize environmental stewardship and responsibility, bringing their operations in accordance with sustainable agricultural methods.

Second, through connecting activist environmental intentions to real environmental behavior, H2 emphasizes how important it is to foster these intentions among farmers. Practical ramifications highlight the importance of initiatives that encourage farmers to collaborate, get involved in the community, and feel like activists with the aim to promote the adoption of sustainable farming practices. Furthermore, H3 and H4 emphasize the critical role that activist and general citizen behavior plays in influencing the overall performance of the environmental system. Achieving EP improves the agriculture paradigms in the perspective of competition and bring sustainability to the EP. Preparing for dynamic environmental changes by agriculture sectors leads them to improved EP. To achieve EP and sustainability of agriculture sector regarding the green environment should consider environmental intentions and behavior of farmers. The environmental behavior that has to be performed by the farmers from the perspective of EP must be made compulsory and environmental-based. For the long run agriculture sector which are involved in the green environmental practices become more competitive and have the ability to utilized the existing resources for the betterment of EP. Therefore, management of agriculture sector must focus on the green activities of farmers for obtaining the updates regarding the ecological stance of various stakeholders in the industry.

Practical ramifications highlight how crucial it is to create and put into place supportive regulatory frameworks. Environmental laws ought to be designed to encourage sustainable behavior, offer precise guidelines, and function as facilitators rather than impediments. Legislators ought to think about establishing a legal framework that supports farmers’ intentions and actions which are environmentally friendly. The combination of these tactics has the potential to significantly enhance the EP of the agriculture industry.

### Limitations and future directions

Instead of making several useful managerial and theoretical implications this research also has few limitations. These limitations are related to the conceptual framework, scope, and other such aspects. These imitations are the guidelines for future researches that will give them direction in their research. Limitation of this study is that it has only discussed the agriculture sector rather than taking a broader view. There are also many other businesses like the SMEs, manufacturing industry, service industry, and so on in which workers environmental intentions and behaviors, and strategic vision of managers can play a significant role in improving the EP.

Another scope that is present for future research that is not covered in the current study is to conduct comparative research due to cultural, regional and agricultural practices. Therefore, cross country analysis can also be done in this regard. Moreover, cultural, regional, and agricultural practices vary widely, which limits the generalizability of results. This suggests that a larger and more varied sample is needed to improve external validity. Furthermore, the effect of responsible environmental intentions and behavior perspective of agriculture sector can be compared with other outcomes instead of EP. Finally, some other moderator variables like institutional and stakeholder’s pressures can also be added to the framework to make it more effective. Therefore, this research has presented a broad scope and opportunities for future researches to be conducted. Lastly, a promising direction for future research that may provide useful answers for raising agricultural sustainability is to look into how precision agriculture and emerging technologies influence people’s intentions and behaviors related to the environment.

## Conclusion

The current study highlighted the role of responsible environmental intentions and behavior towards EP of agriculture industry. Moreover, moderating role of environmental law cognition on the connection between responsible environmental intentions and behavior has also been tested. The current study is based on TPB and NAM, findings revealed that farmer’s citizen and activist environmental intentions have significant impact on how they behave for the protection of natural environment. Furthermore, the findings also confirmed that farmer’s citizen and activist environmental behavior positively predict the EP. Finally, the findings also shows that the moderating role of environmental law cognition extend the TPB and NAM. Ultimately, the aforementioned theories highlight the complex interrelationships that exist at the intersection of environmental intentions, actions, and outcomes within the agricultural sector. According to the research, farmers’ intentions regarding the environment whether broad or activist have a significant impact on how they behave, influencing both the actions of individual citizens and the collective. Furthermore, the research underscores the far-reaching influence of public conduct on the comprehensive environmental outcomes in the agriculture industry.

## Supporting information

S1 Appendix (DOCX)
