# Supplementary material for: Towards environmental performance through responsible environmental intentions and behavior: Does environmental law cognition really matter among Chinese farmers
Source: PLoS One. 2024 Sep 6;19(9):e0308154. doi: 10.1371/journal.pone.0308154 (PMC11379271; doi:10.1371/journal.pone.0308154)
Supplement: S1 Appendix — (DOCX) [file pone.0308154.s001.docx]

**Appendix A:**

| Variables | Items | Constructs |
| --- | --- | --- |
| Environmental Law Cognition (ELC): | ELC1  ELC2  ELC3  ELC4  ELC5  ELC6  ELC7  ELC8  ELC9  ELC10 | Government regulations prioritize ecological defense,  imposing restrictions on tailpipe emissions,  recognizing sound as a pollutant,  Mandating completion of structures post-environmental evaluation,  Linking ecological pollution to legal responsibility,  Ensuring privacy in disclosing harmful actions,  Implementing a manure grant system in China,  penalizing persistent polluters,  Addressing illegal responsibility for severe ecological toxic waste,  Restricting local reporting on community environmental concerns. |
| Responsible Citizen Behavior (RCB): | RCB1  RCB2  RCB3  RCB4  RCB5  RCB6 | Consistent actions include turning off bulbs,  adopting water reduction innovations,  cautious water use,  preference for public transport, utilizing crop waste for soil enrichment,  proper disposal of substance jars,  Recycling plastic bottles. |
| Activist Environmental Behavior (AEB): | AEB1  AE2  AEB3  AEB4 | Active involvement in ecological associations,  support for local defense initiatives,  filing complaints against harmful activities,  Advocating for sustainable agriculture. |
| Responsible Citizens' Environmental Intentions (RCEI): | RCEI1  RCEI2  RCEI3  RCEI4  RCEI5 | Intentions involve favoring continual energy use,  efficient utilization of natural resources on the ranch,  Promoting eco-friendly accommodations, choosing green travel,  Minimizing ranch waste,  Responsibly recycling waste. |
| Activist Environmental Intentions (AEI): | AEI1  AEI2  AEI3  AEI4  AEI5 | Future commitments include safeguarding family from ecological contamination,  Opposing contaminating plans or creations,  Maintaining local ecological efforts, Promoting continual power sources, Embracing natural agriculture. |
| Environmental Performance (EP): | EP1  EP2  EP3  EP4 | Practices impacting overall costs, Overcoming lead time,  Enhancing green products and processes,  Improving reputation contribute to environmental performance. |
